# Change in prevalence and molecular characteristics of isoniazid-resistant tuberculosis over a 10-year period in China

**DOI:** 10.1186/s12879-019-4333-4

**Published:** 2019-08-05

**Authors:** Fengmin Huo, Jie Lu, Zhaojing Zong, Wei Jing, Jin Shi, Yifeng Ma, Lingling Dong, Liping Zhao, Yufeng Wang, Hairong Huang, Yu Pang

**Affiliations:** 1 National Clinical Laboratory on Tuberculosis, Beijing Key laboratory on Drug-resistant Tuberculosis Research, Beijing Chest Hospital, Capital Medical University, Beijing Tuberculosis and Thoracic Tumor Institute, No. 97, Machang, Tongzhou District, Beijing, 101149 China; 2 Beijing Key Laboratory for Pediatric Diseases of Otolaryngology, Head and Neck Surgery, Beijing Pediatric Research Institute, Beijing Children’s Hospital, Capital Medical University, National Center for Children’s Health, Beijing, China

**Keywords:** Tuberculosis, Isoniazid, Drug resistance, inhA, Protionamide

## Abstract

**Background:**

Isoniazid (INH) represents the cornerstone for the treatment of cases infected with *Mycobacterium tuberculosis* (MTB) strains. Several molecular mechanisms have been shown to be the major causes for INH resistance, while the dynamic change of mutations conferring INH resistance among MTB strains during the past decade is still unknown in China.

**Methods:**

In this study, we carried out a comparative analysis of the INH minimal inhibitory concentration (MIC) distribution, and investigate the dynamic change of molecular characteristics among INH-resistant MTB strains between 2005 and 2015.

**Results:**

The proportion of INH resistance (39.0%, 105/269) in 2015 was significantly higher than in 2005 (30.0%, 82/273; *P* = 0.03). Among 269 isolates collected in 2015, 76 (28.3%, 76/269) exhibited high-level INH-resistance (MIC≥32 mg/L), which was significantly higher than that in 2005 (20.5%, 56/273, *P* = 0.04). In addition, a significantly higher percentage of INH-resistant isolates carried *inhA* promoter mutations in 2015 (26.7%) versus that in 2005 (14.6%, *P* = 0.04), while no significant difference was observed in the rates of isolates containing *katG* mutations between 2005 (76.8%) and 2015 (70.5%, *P* = 0.33). Notably, the proportion of MTB isolates with *inhA* mutations (26.7%, 28/105) for patients who had previous exposure to protionamide (PTH) was higher than that for patients who had no previous exposure to PTH (21.4%, 6/28).

**Conclusions:**

In conclusion, our results demonstrated that the proportion of INH-resistant MTB isolates significantly increased during the last decade, which was mainly attributed to an increase of high-level INH-resistant MTB. In addition, prior exposure to PTH may be associated with the increased frequency of INH-resistant tuberculosis strains with *inhA* mutations in China.

**Electronic supplementary material:**

The online version of this article (10.1186/s12879-019-4333-4) contains supplementary material, which is available to authorized users.

## Background

Tuberculosis (TB), is a serious global public health concern, with an incidence of 10.0 million new cases and 1.6 million deaths in 2017 [1]. Despite achieving great progress in curbing the TB epidemic during the last two decades, the efforts to control TB are threatened by the emergence of drug-resistant TB [2], especially multidrug-resistant TB (MDR-TB), defined as the strains resistant to at least isoniazid (INH) and rifampicin (RIF) [3, 4]. As one of the most potent anti-TB drug, isoniazid, together with rifampicin, represents the cornerstone for the treatment of cases infected with RIF-susceptible TB strains [5]. Prior clinical trials have shown that the TB cases with initial INH resistance is at high risk for poor clinical outcomes among the cases receiving the standard first-line therapy regimen, who are more likely to progress to MDR-TB [6, 7]. Hence, the early detection of isoniazid resistance is essential to allow clinicians to adjust initial drug therapy, thereby bringing benefits for patients with INH-resistant TB.

Accurate molecular diagnosis of drug-resistant TB relies on the knowledge of the mechanisms conferring resistance to anti-TB drug [8]. Resistance to isoniazid is a complex process, and several genes, including *katG*, *inhA*, *ahpC*, *kasA* and *ndh*, involve in resistance to INH in MTB [9–11]. Of these targets, two molecular mechanisms have been shown to be the major causes for INH resistance: mutations in *katG* and mutations in the promoter region of *inhA* [11]. The *katG* gene encodes catalase/peroxidase enzyme in MTB, which is responsible for activating the pro-drug isoniazid [9]. As a consequence, the mutations in *katG* lead to the occurrence of INH resistance by the partial or total loss of catalase/peroxidase activity. Multiple studies have shown that *katG* mutations are the most frequent genetic substitutions conferring INH resistance, especially in high-level INH resistant strains [12]. In the latter situation, mutations in the promoter region of *inhA* gene stimulate the overexpression of InhA, the target protein of INH, and cause INH resistance, which are usually associated with low-level resistance [12]. Although approximately 85% of all clinical INH-resistant isolates have the mutations in the two genes, the contribution of these two major mechanisms in INH resistance differ from one geographic region to another, indicating the genetic diversity of INH-resistant MTB strains circulating in various regions [5, 13, 14].

China has been classified as global “hotspot” of drug-resistant TB because of the high prevalence of drug-resistant TB [15]. Although several molecular epidemiological studies have been conducted to describe the molecular characteristics of INH-resistant strains from different regions of China [16–18], the dynamic change of mutations conferring INH resistance among MTB strains during the past decade is still unknown in this country. In a recent study, we identified the increased prevalence of INH resistance among MTB strains between 2005 and 2015 in China [19]. On the basis of our previous findings, we carried out a comparative analysis of the INH minimal inhibitory concentration (MIC) distribution, and investigate the dynamic change of molecular characteristics among INH-resistant MTB strains between 2005 and 2015.

## Methods

### Bacterial strains

Two hundred and eighty MTB strains were respectively selected with a simple random sampling method from pulmonary TB patients seeking health care in Beijing Chest Hospital in 2005 and 2015. Of these 560 strains, 18 failed to grow upon subculture, and the remaining 542 were included for further analysis. All the bacterial cells were stored at − 80 °C in Middlebrook 7H9 broth containing 10% glycerol. Prior to conducting drug susceptibility testing, the strains were recovered on Löwenstein-Jensen (L-J) medium for 4–6 weeks at 37 °C. The demographic information and treatment history were obtained from the medical records.

### Conventional drug susceptibility testing and Mycobacterium species identification

Conventional drug susceptibility testing was performed by the absolute concentration method as previously described [19]. This procedure was performed by inoculating L-J medium supplemented with INH or without drug with MTB. The concentration of INH in medium was 0.2 mg/mL. In addition, the mycobacterium species identification was conducted by biochemical assays with medium containing p-nitrobenzoic acid (PNB) and 2-thiophenecarboxylic acid hydrazide (TCH). Susceptibility to these compounds was defined as the growth of less than 20 colonies on the surface of L-J medium with the corresponding compounds.

### Minimal inhibitory concentration (MIC)

To determine INH MICs of MTB strains, a microplate Alamar blue assay (MABA) was performed as described previously [20]. Briefly, the inoculum was prepared from the 4-week-old cultures grown in L-J medium. The turbidity of the cultures was adjusted to 1.0 McFarland standard. The final inoculum suspension was made by a 1:20 dilution in Middlebrook 7H9 broth containing 10% OADC, and then 0.1 mL was delivered to the wells of the 96-well plate with serial two-fold dilutions of INH in 100 μL of 7H9 broth. After 7 days of incubation, 70 μL of Alamar Blue solution was added to each well, and incubated for another 24 h at 37 °C. A change from blue to pink indicates the bacterial growth. MIC was defined as the lowest concentration of INH that inhibited the color change from blue to pink. INH concentrations were doubling dilutions from 0.063 to 32 μg/mL. The INH resistance was defined as an MIC of ≥0.5 μg/mL, which was referred to the previous literature [21]. In addition, low-level resistance to INH was defined as an MIC of ≤2 μg/mL; moderate-level resistance was defined as an MIC ranging from 4 to 16 μg/mL; high-level resistance was defined as an MIC of ≥32 mg/L. *M. tuberculosis* H37Rv (ATCC 27249) was tested in both rounds as a quality control strain.

### DNA amplification and sequencing

Crude genomic DNA was extracted from freshly cultured bacteria using the boiling lysis method as previously reported [19]. The partial fragments of *katG* and the promoter region of *inhA* gene were amplified with the primers as listed in the Additional file 1: Table S1. The PCR mixture was prepared in a volume of 50 μL containing 25 μL 2 × GoldStar Best MasterMix (CWBio, Beijing, China), 2 μL of DNA template and 0.2 μM of each primer set. The PCR products were sent to Qingke Company for DNA sequencing service. DNA sequences were aligned with the corresponding sequences of the reference *M. tuberculosis* H37Rv strains using BioEdit software (http://www.mbio.ncsu.edu/bioedit/bioedit.html).

### Statistical analysis

The rates of categorical variables between groups were compared using the Chi-square test (Fisher’s exact test when the expected number was less than five). Statistical analysis was performed in SPSS 17.0 software (SPSS Inc., USA). Difference with *P* < 0.05 was considered to indicate significant difference for chi-square test, while for the paired comparisons, the difference was considered as significant if *P* value was less than false discovery rate (FDR) to reduce the false positive results [21].

## Results

### Factors associated with the increased prevalence of INH-resistant TB

A total of 542 MTB isolates were enrolled in this study, including 273 isolates (50.4%) collected in 2005 and 269 isolates (49.6%) collected in 2015. The proportion of INH resistance (39.0%, 105/269) in 2015 was significantly higher than in 2005 (30.0%, 82/273; *P* = 0.03). Similar, RIF resistance was identified in 36.4% (98/269) of MTB isolates in 2015, which was significantly higher than that in 2005 (28.2%, 77/273, *P* = 0.04). In addition, there was no significant difference in the proportion of MDR-TB between 2005 (24.2%, 66/273) and 2015 (31.2%, 84/269, *P* = 0.07). We further analysed the distribution of INH-resistant TB with respect to sex, age and treatment history. As shown in Table 1, the proportion of male INH-resistant cases in 2015 (65.7%, 69/105) was significantly higher than in 2005 (54.9%, 45/82; *P* = 0.01). Similarly, INH-resistance were found in 38 (22.5%) of 169 new cases in 2005, with a significantly higher proportion of such cases observed in 2015 (33.3%, 63/189; *p* = 0.02). In contrast, the distributions of INH-resistant cases according to age had no statistically significant differences between 2005 and 2015 (*P* > 0.05).

**Table 1:** Distribution of INH-resistant MTB isolates among demographic and clinical characteristics between 2005 and 2015 in this study

Characteristics			No. (%) of isolates		*P* value	OR 95% CI
	2005 (*n* = 273)		2015 (*n* = 269)			
	INH-R (*n* = 82)	INH-S	INH-R	INH-S		
		(*n* = 191)	(*n* = 105)	(*n* = 164)		
Sex						
Men	45(54.9)	123(64.4)	69(65.7)	103(62.8)	0.01	1.83 (1.16–2.89)
Women	37(45.1)	68(35.6)	36(34.3)	61(37.2)	0.78	1.08 (0.61–1.93)
Age group (years)						
<25	9(11.0)	22(11.5)	9(8.6)	19(11.6)	0.80	1.16(0.38–3.51)
25–44	34(41.5)	72(37.7)	42(40.0)	54(32.9)	0.09	1.65(0.93–2.92)
45–64	28(34.1)	64(33.5)	39(37.1)	59(36.0)	0.18	1.51 (0.83–2.76)
>64	11(13.4)	33(17.3)	15(14.3)	32(19.5)	0.47	1.41(0.56–3.52)
Treatment History						
New case	38(46.3)	131(68.6)	63(60.0)	126(76.8)	0.02	1.72(1.08–2.76)
Re-treated	44(53.7)	60(31.4)	42(40.0)	38(23.2)	0.17	1.51(0.84–2.71)

### MICs and profiling of genetic mutations of INH-resistant MTB isolates

**Fig. 1:**
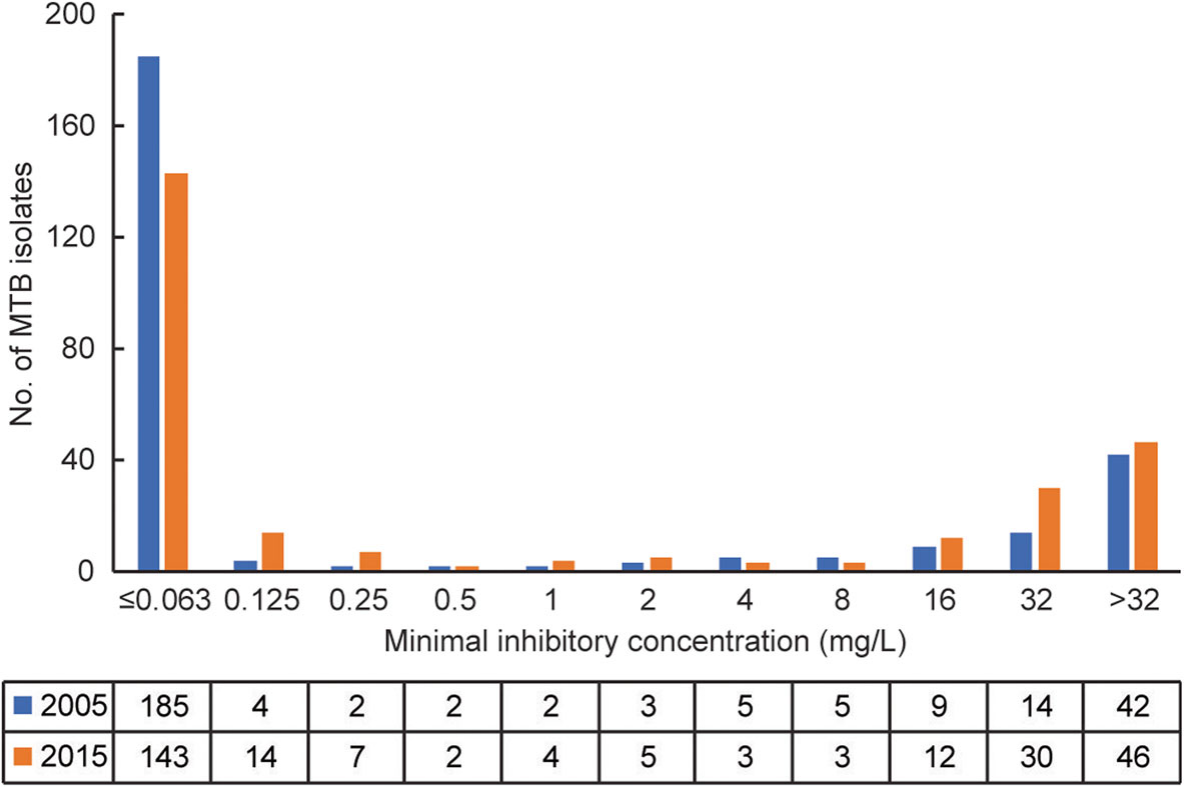
Distributions of the INH MICs of the MTB isolates enrolled in this study

The distribution of MTB isolates with different MIC values was summarized in Fig. 1. Overall, 67.8% (185/273) and 53.2% (143/269) of MTB isolates tested had MIC values no more than 0.063 mg/L in 2005 and 2015, respectively. Among 269 isolates collected in 2015, 76 (28.3%, 76/269) exhibited high-level INH-resistance (MIC≥32 mg/L), which was significantly higher than that in 2005 (20.5%, 56/273, *P* = 0.04). We further investigated genetic mutation profiles among INH-resistant MTB isolates. As shown in Table 2, mutations within *katG* and promoter of *inhA* were found in 82.9% (68/82) of INH-resistant isolates in 2005 and 81.9% (86/105) in 2015, respectively. The *katG* mutation alone was observed most frequently, accounting for 56 in 2005 and 58 in 2015. In addition, *inhA* promoter region T-8C mutation alone was only identified in 4 isolates collected in 2015. Statistical analysis revealed a significantly higher percentage of INH-resistant isolates carrying *inhA* promoter mutations in 2015 (26.7%) versus that in 2005 (14.6%, *P* = 0.04), while no significant difference was observed in the rates of isolates containing *katG* mutations between 2005 (76.8%) and 2015 (70.5%, *P* = 0.33).

**Table 2:** Comparison of mutant profiles in *katG* and *inhA* among INH-resistant isolates between 2005 and 2015

Mutation type	No. of INH-resistant isolates with different mutations (%)		*P* value
	2005 (*n* = 82)	2015 (*n* = 105)	
Single Mutation			
*inhA − 8*	0(0.0)	4(3.8)	0.13
*inhA − 15*	5(6.1)	8(7.6)	0.69
*katG 315*	56(68.3)	58(54.3)	0.07
Total	61(74.4)	70(65.7)	0.25
Double Mutations			
*inhA* − 8 + *katG* 315	3(3.7)	4(3.8)	1.00
*inhA* − 15 + *katG* 315	4(4.9)	12(11.4)	0.11
Total	7(8.5)	16(15.2)	0.17
With mutations			
*inhA*	12(14.6)	28(26.7)	0.04
*katG*	63(76.8)	74(70.5)	0.33
Without mutation	14(17.1)	19(18.1)	0.86

### Correlation between phenotypic resistant level and mutations conferring INH resistance

**Fig. 2:**
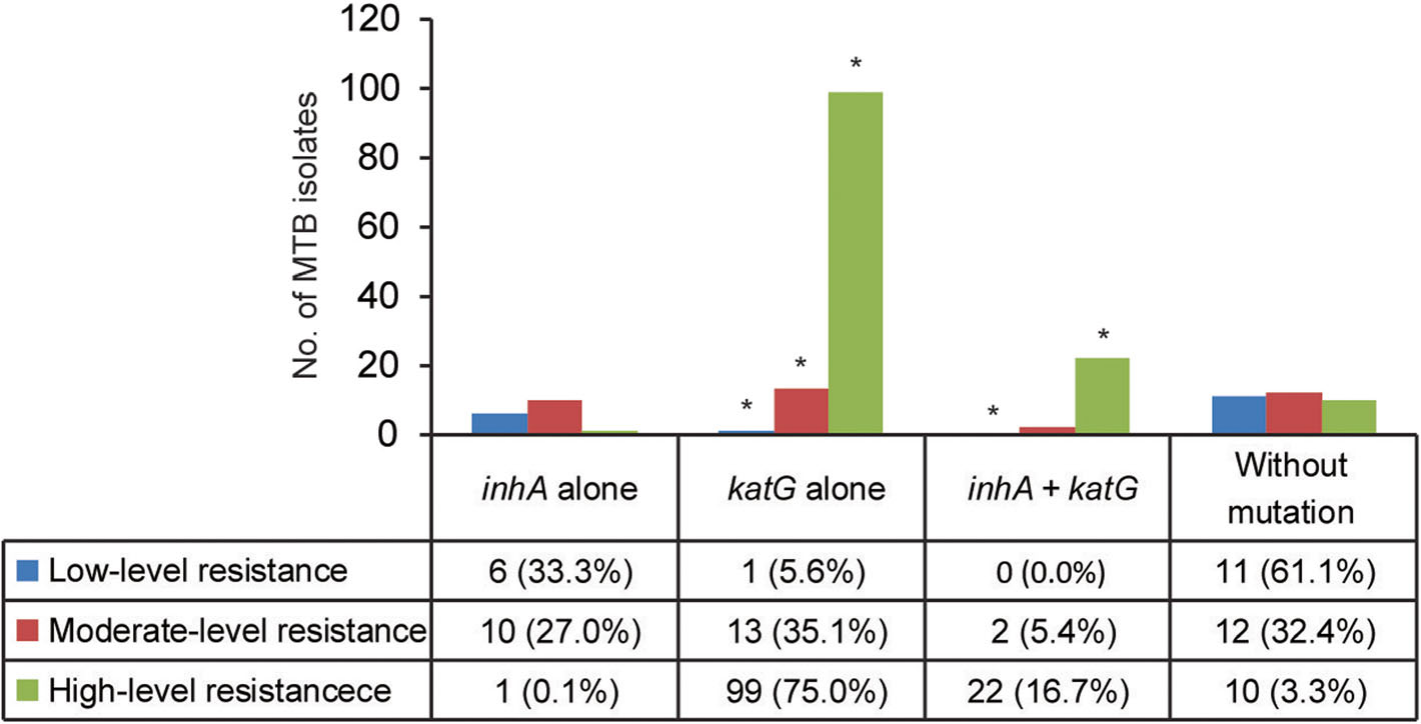
Correlation between phenotypic resistant level and mutations conferring INH resistance. Low-level resistance: 0.5 ≤ MIC ≤2 mg/L; moderate-level resistance: 4 ≤ MIC ≤16 mg/L; high-level resistance: MIC ≥32 mg/L; asterisk represents significant differences [*P* value is less than the false discovery rate (FDR) 0.017] between the group with various mutation types and the group without mutations

We further compared the distribution and resistance levels to INH of MTB isolates harboring different mutation types. The results are summarized in Fig. 2. Generally, the highest proportion of MTB isolates with high-level resistance was identified in those with mutations in both *inhA* and *katG*, which was significantly higher than that of isolates without mutation. Similarly, *katG* mutation alone correlated with high-level resistance and the moderate-level resistance, while was present significantly less often in the low-level resistant group compared to the group without mutation. In addition, there was no significant differences in the distribution and resistance levels to INH between group with *inhA* mutation alone and group without mutation (*P* > 0.05).

### Distribution of MTB isolates with *inhA* mutations

We further analyzed the distribution of MTB isolates with *inhA* mutations according to patients’ characteristics, so that to identify risk factors associated with the increased rate of MTB isolates harboring *inhA* promoter mutation in 2015. The results are summarized in Table 3. Notably, the proportion of MTB isolates with *inhA* mutations (26.7%, 28/105) for patients who had previous exposure to protionamide (PTH) was higher than that for patients who had no previous exposure to PTH (21.4%, 6/28). On the contrary, no significant different in prevalence was observed among patient sex, age and various drug exposure history groups (*P* > 0.05).

**Table 3:** Factors associated with MTB strains harboring *inhA* mutation in 2015

Characteristics	No. of INH-resistant isolates (%)		*P* value	OR (95% CI)
	With *inhA* mutation (*n* = 28)	Without *inhA* mutation (*n* = 77)		
Sex				
Men	18(64.3)	51(66.2)	0.85	1.09(0.44–2.70)
Women	10(35.7)	26(33.8)		1.00(Ref)
Age group (years)				
<25	3(10.7)	6(7.8)	0.39	0.47(0.10–2.30)
25–44	8(28.6)	34(44.2)		1.00(Ref)
45–64	12(42.9)	27(35.1)	0.22	0.53(0.19–1.48)
>64	5(17.9)	10(13.0)	0.29	0.47(0.13–1.76)
Treatment History				
New case	16(35.7)	47(79.2)		1.00(Ref)
Re-treated	12(64.3)	30(20.8)	0.72	0.85(0.35–2.05)
Previous drug exposure				
INH	12(77.8)	30(26.0)	0.72	0.85(0.35–2.05)
RIF	12(77.8)	30(26.0)	0.72	0.85(0.35–2.05)
EMB	12(77.8)	30(26.0)	0.72	0.85(0.35–2.05)
PZA	12(77.8)	30(26.0)	0.72	0.85(0.35–2.05)
SLID	8(32.1)	19(15.6)	0.69	0.82(0.31–2.16)
FQ	10(39.3)	22(20.8)	0.48	0.72(0.29–1.80)
PTH	6(21.4)	4(5.2)	0.02	0.20(0.05–0.78)

## Discussion

Drug-resistant TB emerges as a major threat to effective TB control and the attainment of the 2035 global targets in China [22]. Our data demonstrate that the proportion of INH-resistant MTB isolates significantly increased during the last decade [19], which was mainly attributed to an increase of high-level INH-resistant MTB. Consistency with our results, the increasing prevalence of drug-resistant TB has been noted by several recent reports in China [19, 23]. There is no doubt that the use of anti-TB drugs can affect the development of drug-resistant tubercle bacillus. Considering that INH is one of the most important first-line drug administered by TB patients during intensive and continuation phases, the prolonged exposure duration would facilitate the occurrence of INH-resistant bacteria. In addition, the fact that the choice of dose for antibiotic treatment can select various levels of drug resistance [24]. Although the dosage of INH for treatment of TB patients is not changed, the increasing prescription of p-aminosalicylic acid (PAS) [25], a competitive substrate that decreases in vivo acetylation and dehydrazination of INH, may elevate the INH concentration in patients receiving a combination of INH and PAS [26], thereby accelerating the emergence of high-level INH resistance.

Several recent reports have examined the contribution of *katG* and *inhA* promoter mutations in INH-resistant MTB isolates, and shown significant geographic diversity across regions [27]. In the present study, mutations in *inhA* alone were observed in 11.4% resistant isolates in 2015. The frequency of such mutants conferring INH resistance is similar to that in Chongqing (10%) [28] and that in China (11%) [16], although it is lower than that in Philippines (22%) [29] and that in South Africa (30%) [30], and higher than that in Lithuania (3.4%) [5] and that in Poland (4%) [31]. The regional differences in the frequencies of mutations associated with INH resistance may reflect the diversity in molecular characteristics of predominant MTB isolates circulating in geographically distinct areas, and also provide hints for the development of molecular-based diagnostic tests.

In line with previous studies, in vitro experimental data of this study indicated that *katG* codon 315 mutations are associated with high-level resistance to INH, while mutations in *inhA* regulatory region confer low-level resistance to INH [12]. We also observed that 3.3% of INH-resistant isolates without *katG* and *inhA* mutation exhibited high-level resistance. Generally, efflux pump and other natural mechanisms always medicate low-level resistance, while the high-level resistance is due to mutations in the target genes [3]. Hence, we speculate that mutations in other candidates, such as *oxyR-ahpC* intergenic region, *kasA*, and *ndh*, may be responsible for these isolates exhibiting high-level resistance [11]. Further experimental data will extend our knowledge on molecular mechanisms of novel genes involving INH resistance in MTB.

Another interesting finding of our report was that the prevalence of INH-resistant isolates carrying mutations in *inhA* regulatory region was significantly increased between 2005 and 2015. In addition, our analysis results showed a significant association between *inhA* promoter mutations and previous PTH exposure. A serial of previous studies has reported that *inhA* promoter mutations not only cause INH resistance, but they also confer cross-resistance to ethionamide (ETH) and PTH [32]. In most cases, PTH is used as a subsequent choice for the patients suffering treatment failure with the INH-containing first-line regimen. More importantly, a recent survey indicates that there was a significant increasing trend for PTH prescribing for TB patients in China during the past years [33]. Hence, the preexisting *inhA* promoter mutations may facilitate successful adaptation to the PTH exposure at low fitness cost for INH-resistant MTB isolates. Despite strong evidence linking *inhA* promoter mutations and ETH/PTH resistance for MTB, these mutations may play a compensatory role in increasing their fitness due to the absence of experimental data in the present study. Therefore, further in vitro experiments are urgently needed to clarify the selective advantage of these mutants during exposure to ETH/PTH.

Our study has several important hints for clinical use of INH in the treatment of TB patients. On one hand, high-dose INH has been included in the WHO guidelines for the treatment of MDR patients with low-level INH resistance [34]. However, the increasing prevalence of high-level INH resistance among MTB isolates may impair the effectiveness of high-dose INH for these patients in China. On the other hand, considering that *inhA* promoter mutations generate additional cross-resistance to PTH. The inclusion of PTH in the regimen should be used for the treatment of INH-resistant patients without *inhA* promoter mutation. Taken together, these data underscore the need to clarify the specific resistance mutations for INH-resistant TB cases in order to guide clinicians in making therapeutic decisions appropriately.

## Conclusion

In conclusion, our results demonstrated that the proportion of INH-resistant MTB isolates significantly increased during the last decade, which was mainly attributed to an increase of high-level INH-resistant MTB. In addition, prior exposure to PTH may be associated with the increased frequency of INH-resistant tuberculosis strains with *inhA* mutations in China. Therefore, our findings highlight the need to clarify the specific resistance mutations for INH-resistant TB cases in order to guide clinicians in making therapeutic decisions appropriately.

## Additional file

Additional file 1: **Table S1.** Primers used for DNA sequencing. (DOCX 15 kb)

## Data Availability

The datasets used and/or analysed during the current study available from the corresponding author on reasonable request.

## Abbreviations

ETH: ethionamide
FDR: false discovery rate
INH: Isoniazid
L-J: Lowenstein-Jensen
MABA: microplate Alamar blue assay
MDR-TB: multidrug-resistant TB
MIC: minimal inhibitory concentration
MTB: *Mycobacterium tuberculosis*
PAS: para-aminosalicylic acid
PNB: p-nitrobenzoic acid
PTH: protionamide
RIF: rifampicin
TB: tuberculosis
TCH: 2-thiophenecarboxylic acid hydrazide
