# Supplementary material for: Change in prevalence and molecular characteristics of isoniazid-resistant tuberculosis over a 10-year period in China
Source: BMC Infect Dis. 2019 Aug 5;19:689. doi: 10.1186/s12879-019-4333-4 (PMC6683513; doi:10.1186/s12879-019-4333-4)
Supplement: Supplementary file 1 — Table S1. Primers used for DNA sequencing. (DOCX 15 kb) [file 12879_2019_4333_MOESM1_ESM.docx]

Table S1 Primers used for DNA sequencing

| Gene | Orientation | Oligonuc leotide sequence (5'–3') |
| --- | --- | --- |
| KatG-F | Forward | GCTCGGCGATGAGCGTTAC |
| KatG-R | Reverse | CTCGTAGCCGTACAGGATCTCG |
| InhA-F | Forward | TCGCAGCCACGTTACGCTC |
| InhA-R | Reverse | CCAGCCGCTGTGCGATC |
